# Kinetics of Severity Biomarkers and Immunological Features of Methylprednisolone Therapy for Severe COVID-19 Patients

**DOI:** 10.3389/fimmu.2022.758946

**Published:** 2022-03-08

**Authors:** Qinghong Fan, Kai Deng, Huang Huang, Ruiying He, Xizi Deng, Yun Lan, Yizhou Tan, Weilie Chen, Yaping Wang, Xilong Deng, Fengyu Hu, Feng Li

**Affiliations:** ^1^ Guangzhou Eighth People’s Hospital, Guangzhou Medical University, Guangzhou, China; ^2^ Guangzhou Laboratory, Bio-Island, Guangzhou, China

**Keywords:** methylprednisolone, severe COVID-19, C-reactive protein, cytokine release storm, “core signature” cytokines

## Abstract

In contrast to dexamethasone, the clinical efficacy of methylprednisolone (MP) remains controversial, and a systems biology study on its mechanism is lacking. In this study, a total of 38 severe COVID-19 patients were included. The demographics, clinical characteristics, and severity biomarkers including C-reactive protein (CRP), d-dimer, albumin, and Krebs von den Lungen 6 of patients receiving MP (n=26, 40 mg or 80 mg daily for 3-5 days) and supportive therapy (n=12) were compared. Longitudinal measurements of 92 cytokines in MP group from admission to over six months after discharge were performed by multiplex Proximity Extension Assay. The results showed that demographics, baseline clinical characteristics were similar in MP and non-MP groups. No death occurred and the hospital stays between the two groups were similar. Kinetics studies showed that MP was not better than supportive therapy at improving the four severity biomarkers. Cytokines in MP group were characterized by five clusters according to their baseline levels and responses to MP. The immunological feature of severe COVID-19 could be defined by the “core signature” cytokines in cluster 2: MCP-3, IL-6, IFN-γ, and CXCL10, which strongly correlated with each other and CRP, and are involved in cytokine release storm. The “core signature” cytokines were significantly upregulated at baseline and remained markedly elevated after MP treatment. Our work showed a short course of MP therapy could not rapidly improve the immune disorders among severe COVID-19 patients or clinical outcomes, also confirmed “core signature” cytokines, as severity biomarkers similar to CRP, could be applied to evaluate clinical treatment effect.

## Introduction

The coronavirus disease 2019 (COVID-19), which is caused by highly contagious severe acute respiratory syndrome coronavirus 2 (SARS-CoV-2), has caused over 200 million infections and 4.2 million deaths as of August 11, 2021 (1). A national-wide survey including 72,314 confirmed COVID-19 cases in mainland China showed that 19% of patients developed severe or critical disease, and the mortality rate in the critically ill was extremely high (49%) (2). Similarly, the mortality rates among severe and critical patients in the US ranged from 50% to 65% (3–5). Thus, prevention of disease progression and rescue of deteriorating patients play critical roles in COVID-19 treatment.

Severe COVID-19 patients frequently develop acute respiratory distress syndrome (ARDS), with extra clinical manifestations including thromboembolic complications, dysfunctions of central or peripheral nervous system, and elevations of C-reactive protein (CRP) and IL-6 (6). Ravaging SARS-CoV-2 infection typically results in overactivation of innate and adaptive immune responses, the term “cytokine release storm (CRS)”, which could be caused by various fatal infections, complications of malignant tumors, and autoimmune diseases, and characterized by elevations of various inflammatory cytokines, e.g. IL-6, IL-10, IL-8, and CXCL10 (7–13). In CRS, all the cytokines reflecting activated type 1 antiviral cellular immune response (IFN-γ), type 2 anti-helminths cellular immune response (IL-5, IL-13), and type 3 antifungal cellular (IL-17, IL-22) were elevated (12). Therefore, restoring immune balance and suppressing markedly elevated inflammatory cytokines are critical for curing severe COVID-19 patients and lowering mortality.

By now at least four therapeutics for severe COVID-19 have been recommended by National Institutes of Health (NIH): Remdesivir (direct-acting antiviral), dexamethasone (long-acting corticosteroid), tocilizumab (IL-6 receptor blocker), and baricitinib (Janus kinase 1 and 2 inhibitor) (14). Notably, the last three therapeutics aim to mitigate the immune response and prevent a hyperinflammatory state, which is involved in CRS. In contrast to dexamethasone, whose benefit had been confirmed in “RECOVERY” clinical trial (15), evidence to support the use of other corticosteroids such as methylprednisolone (MP) was not so robust, and contradictory results emerged (16). Ramiro et al. reported that high-dose MP combined with tocilizumab facilitated the recovery of COVID-19 patients with CRS, and significantly reduced the mortality rate (17), and another study showed a prolonged MP administration was associated with lower mortality in severe COVID-19 patients (18). In contrast, a meta-analysis conducted by World Health Organization suggested MP was not significantly associated with lower mortality in critically ill patients (19), and a phase II clinical trial reported a short course of MP could not reduce the mortality in hospitalized COVID-19 patients (20). Despite these discrepancies, a comprehensive and longitudinal evaluation of the kinetics of inflammatory cytokines among COVID-19 patients who received MP is still lacking, which could provide a valuable tool to understand the role of MP in curing severe COVID-19 and also fill the knowledge gaps on the immunological features of this population. In this retrospective study, we performed a systematic assessment of MP effect by use of a set of well-studied COVID-19 severity biomarkers including CRP, d-dimer, albumin (Alb), and Krebs von den Lungen 6 (KL-6) (21), as well as a broad spectrum of inflammatory cytokines from admission to over six months after discharge.

## Materials and Methods

### Study Population

Guangzhou Eighth People’s Hospital was the treatment center for COVID-19 in Guangzhou, and local patients would be firstly enrolled here according to prevention policy. This retrospective study was approved by Guangzhou Eighth People’s Hospital Ethics Committee (No. 202001134), and written informed consent was obtained from patients. COVID-19 severity was evaluated according to Diagnosis and Treatment Protocol for Novel Coronavirus Pneumonia (Eighth edition, General Office of National Health Commission, China), and “severe patients” here refers to both severe and critically ill patients meeting any of the following: (1) shortness of breath, respiratory rate≥30 times/min; (2) in the resting state, during inhalation, the oxygen saturation is ≤93%; (3) arterial partial pressure of oxygen (PaO2)/inhaled oxygen concentration (FiO2)≤300mmHg (1mmHg=0.133kPa); (4) The clinical symptoms are progressively worsening, and lung imaging shows that within 24 to 48 hours the lesion has progressed significantly >50%. In total, 38 severe COVID-19 patients hospitalized at Guangzhou Eighth People’s Hospital from January 20, 2020, to March 13, 2020, were included, representing most local severe COVID-19 patients in 2020 (88.4%, 38/43), 5 severe patients were not included because they were transferred and treated elsewhere. Among the severe COVID-19 patients, 26 of them received sodium succinate MP (40 mg or 80 mg) once daily for 3-5 days, and the other 12 patients received supportive therapy without MP treatment. Demographic, clinical characteristics, and laboratory findings were collected from electronic medical charts. Healthy control (n=9) consisted of 3 males and 6 females, and the median age was 41 years.

### Sample Collection and Plasma Cytokine Measurement

The time points of sample collection include before, during, and after MP treatment during hospitalization, and over six months after discharge. The samples of nine healthy individuals, who had a median age of 41 years (interquartile range [IQR]: 35-44 years), were also included as a control. Anticoagulated peripheral blood was collected from COVID-19 patients and plasma was separated by centrifugation at 3000 rpm for 6 min at room temperature, aliquoted, and stored at −80°C until use. Plasma samples were inactivated with 1% Triton X-100 in phosphate-buffered saline (v/v) incubation at room temperature for 2 hours. A total of 92 inflammation-related cytokines were measured using Olink^®^ inflammation panel of multiplex Proximity Extension Assay (PEA) according to the manufacturer’s instructions (www.olink.com, **Table S1**). Briefly, the target cytokine was recognized by two paired oligonucleotide-conjugated antibodies, and the templates, that the paired oligonucleotide sequences, were amplified by quantitative real-time PCR. Data analysis was performed with normalized protein expression (NPX), which was generated on a Log2 scale where a larger number represented a higher level of target cytokine in the sample. The data were pre-processed by Olink^®^ using NPX Manager software. The complete list of cytokines and their abbreviations are shown in **Table S1**.

### Statistics Analysis

Continuous variables were expressed as median (IQR) or mean ± SEM. Categorical variables were summarized as the counts and percentages in each category. Student’s t-test tests or Mann-Whitney tests were applied to continuous variables as appropriate. Pearson’s rank correlation was used to explore the correlations between different parameters, p<0.05 was considered statistically significant. Statistical analysis was performed with IBM SPSS Statistics 25. Graphic representations were performed with GraphPad Prism 8 software. Full-spectrum heatmap of 92 inflammatory cytokines and the graphical representation of correlations were performed with “pheatmap” and “corrplot” packages of R studio, respectively.

## Results

### Baseline Characteristics of Severe COVID-19 Patients

Guangzhou Eighth People’s Hospital has been the only designated treatment center to manage COVID-19 patients since the epidemic began. A total of 43 severe COVID-19 patients were enrolled in 2020 and 5 of them were transferred elsewhere shortly after admission, and the remaining 38 severe COVID-19 patients were treated here till discharge and therefore included in our study. Among them, 26 were treated with 40 mg or 80 mg MP per day within several days of admission (median [IQR]= 1[1-3] days) for 3-5 days, and 12 severe patients only received supportive therapy and were defined as “non-MP group”. The median ages of MP and non-MP groups were 58.0 and 59.5 years, respectively (p=0.698, **Table 1**). The gender distribution and comorbidity proportion of the two groups were similar (p=1.000). Upon admission, 92.3% (24/26) of patients in MP group and 100% (12/12) of patients in non-MP group received oxygen support (p=1.000). Besides, the proportions of patients receiving anticoagulant therapy in MP and non-MP groups were similar (p=0.984). The well-studied COVID-19 severity biomarkers, serum CRP (22, 23) and d-dimer (24, 25), were elevated in MP and non-MP groups on admission (CRP>10 mg/L, d-dimer>1000 µg/L), and albumin (26, 27) was decreased (Alb<40 g/L). The three biomarkers on admission were not significantly different between MP and non-MP groups (p>0.05, **Table 1**). Recently we and other groups identified serum KL-6 as a novel biomarker of COVID-19 severity (21, 28), here we also determined the KL-6 level of severe patients on admission and found it was similar in MP and non-MP groups (420.9 *vs* 407.8 U/mL, p=1.000). In all, the demographics, baseline clinical characteristics, and severity biomarkers were not significantly different between MP and non-MP groups.

**Table 1:** Demographics and baseline characteristics of severe COVID-19 patients.

Characteristic	MP treated (n=26)	Non-MP treated (n=12)	p value
age, years	58.0 (50.8-66.0)	59.5 (52.0-63.0)	0.698
male	16 (61.5%)	8 (66.7%)	1.000
oxygen support	24 (92.3%)	12 (100%)	1.000
anticoagulant	7 (26.9%)	4 (33.3%)	0.984
comorbidity, ≥1	22 (84.6%)	10 (83.3%)	1.000
CRP, mg/L	39.8 (19.7-75.5)	34.7 (25.5-47.4)	0.545
d-dimer, µg/L	1670 (1105-2220)	1860 (1645-3460)	0.149
Alb, g/L	34.8 (31.9-38.2)	35.4 (33.5-37.1)	0.782
KL-6, U/mL	420.9 (245.8-749.6)	407.8 (306.8-457.1)	1.000

Continuous variables were expressed as medians (interquartile). Categorical variables were summarized as the counts and percentages in each category. Student’s t test and Mann–Whitney U test were applied to continuous variables as appropriate, and χ^2^ test was used for categorical variables. CRP, C-reactive protein; Alb, albumin; KL-6, Krebs von den Lungen 6. The upper limit is 10 mg/L for CRP, and 1000 μg/L for d-dimer; the lower limit of Alb is 40 g/L.

### Clinical Outcome and Kinetics of COVID-19 Severity Biomarkers

**Figure 1:**
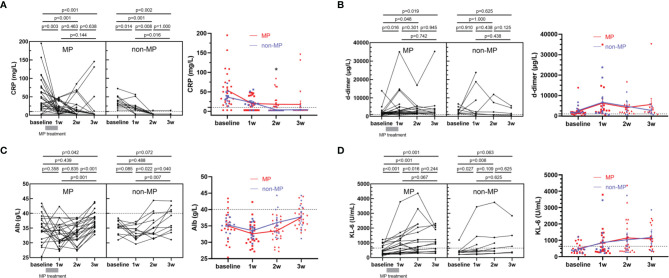
Kinetics of COVID-19 severity biomarkers including CRP **(A)**, d-dimer **(B)**, Alb **(C)**, and KL-6 **(D)** in MP (n=26) and non-MP (n=12) groups. Data are presented as mean ± standard error. Student’s paired or unpaired tests, or Mann-Whitney tests were applied as appropriate. A dotted line indicates the upper limit of CRP (10 mg/L) and d-dimer (1000 μg/L), the lower limit of Alb (40 g/L). *p < 0.05.

There was no death among severe COVID-19 patients during the study period, and 13 patients (50.0%) in MP group and 9 (75.0%) in non-MP group were transferred to ICU (p=0.147), and 11 (42.3%) in MP and 6 (50.0%) in non-MP groups received mechanical ventilation (p=0.658). The average hospital stays in MP and non-MP groups were 26.2 days and 22.2 days, respectively (p=0.160). Given that no death occurred in the cohorts, we used COVID-19 severity biomarkers including CRP, d-dimer, Alb, and KL-6 as alternatives to mortality to evaluate the clinical efficacy of MP. The CRP level in MP group was markedly elevated at baseline (median=39.8 mg/L, **Figure 1A** left panel), and significantly decreased after MP treatment (approximately 1 week after admission, median=17.0 mg/L, p=0.003), and then progressively declined over time till discharge (**Figure 1A** right panel). For patients in non-MP group, the median level of CRP significantly decreased from 34.7 mg/L at baseline to 19.1 mg/L at 1 week after admission (p=0.014), and continuously decreased to 3.0 mg/L at 2 weeks after admission (p=0.008). Since the hospital stays were similar between MP and non-MP groups, we compared CRP in the two groups at the same time points, and found their CRP levels at baseline, 1 week, and 3 weeks after admission were not significantly different; in contrast, CRP at 2 weeks after admission was lower in non-MP group than that in MP group (median [IQR]: 3.0 [3.0-3.0] *vs* 3.0 [3.0-23.5] mg/L, p=0.030, **Figure 1A** right panel). The level of d-dimer in MP group significantly increased after MP treatment (1w *vs* bassline=2950 *vs* 1670 µg/L[median], p=0.016) and remained elevated before discharge; d-dimer in non-MP group was also elevated at baseline and did not vary significantly during hospitalization (**Figure 1B**). Alb in MP group was decreased at baseline (<40 g/L) and started to increase at 2 weeks after admission (2w *vs* 3w=33.5 *vs* 37.1 g/L [median], p<0.001), while Alb in non-MP group increased as early as 1 week after admission (**Figure 1C**). KL-6 continuously and significantly increased in MP and non-MP groups within 2 weeks and 1 week after admission, respectively (**Figure 1D**). Despite the differences in kinetics, the levels of d-dimer, Alb, and KL-6 were not significantly different at the same time points between MP and non-MP groups. Given that the demographics and baseline variables were similar between MP and non-MP groups, these data suggested MP administration for severe COVID-19 was not better than supportive therapy at improving clinical outcome and severity biomarkers.

### Longitudinal Changes in Inflammatory Cytokines in MP Group

**Figure 2:**
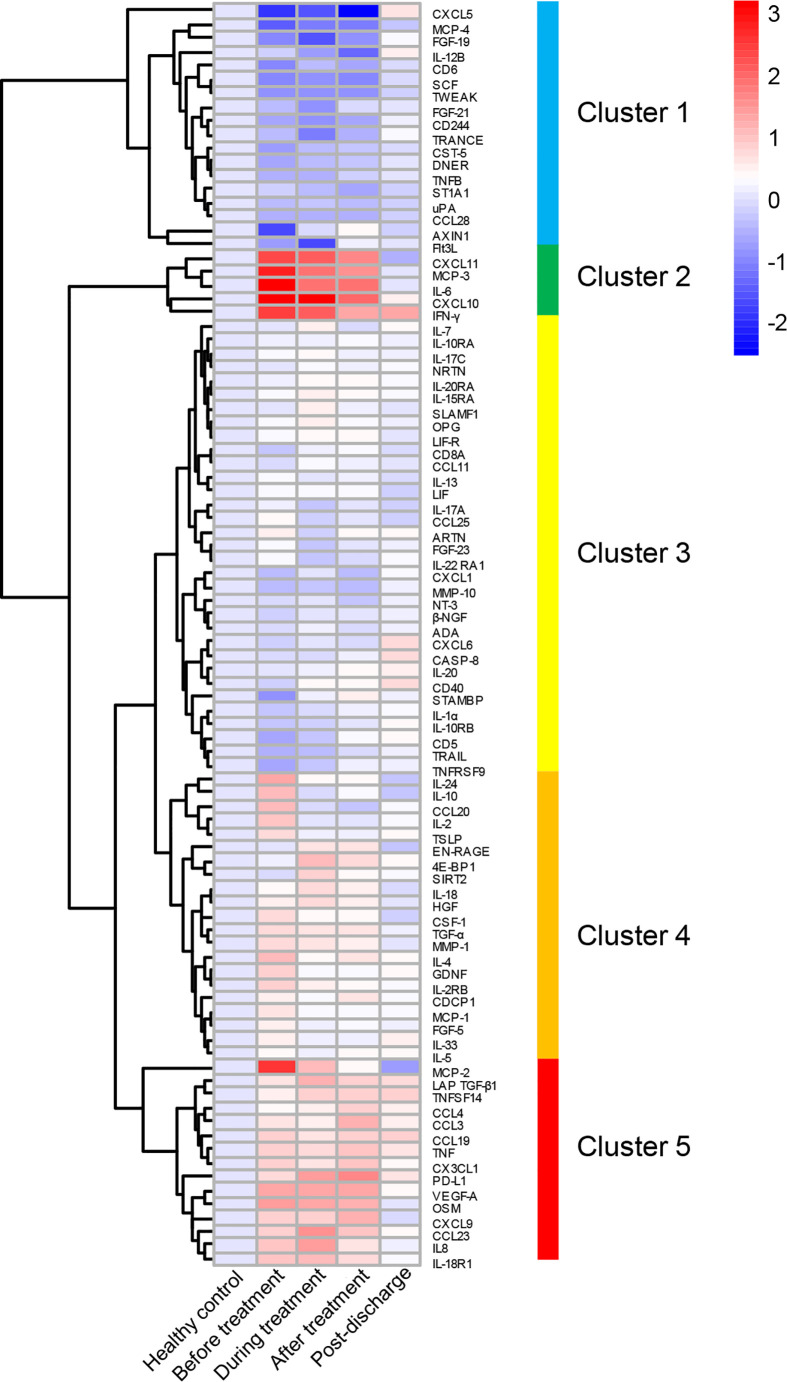
Longitudinal changes in inflammatory cytokines in MP group. The relative levels of 92 cytokines were depicted with normalized protein expression (NPX) and shown in heatmap, 92 cytokines were divided into five clusters as shown in heatmap. The full spectrum heatmap was performed with “pheatmap” packages of R studio.

One of the distinctive features of severe COVID-19 is the excessive elevation of multiple cytokines such as IL-6, IL-10, and IFN-γ (29–35). Upon admission, the severe patients displayed a marked elevation in acute-phase reactants including CRP and d-dimer, and hypoalbuminemia (**Table 1** and **Figure 1**), suggesting ongoing immunological abnormalities (34). Since MP did not show greater benefit among severe COVID-19 patients compared with supportive therapy, we conducted a longitudinal analysis of immune inflammatory indexes in MP group from admission to over six months after discharge by simultaneously evaluating 92 immune inflammation-related pathway cytokines using the PEA method, aiming to explore the immunological features of this population, as well as the effect of MP on inflammatory cytokines. We observed a marked elevation of various cytokines including pro-inflammatory cytokines (IL-6, IL-8, and IFN-γ), immunostimulatory chemokines (CXCL10, CXCL11, and CX3CL1), an inflammatory chemokine MCP-3, and a pro-inflammatory mediator Oncostatin M (OSM) in MP group at baseline compared to healthy control (**Figure 2**). Most of the cytokines, such as IL-6, IFN-γ, and CXCL10, were linked to CRS (34). In contrast, cytokines CD6 and Delta and Notch-like epidermal growth factor-related receptor (DNER) were significantly decreased at baseline (p=0.021 and p=0.007, respectively).

**Figure 3:**
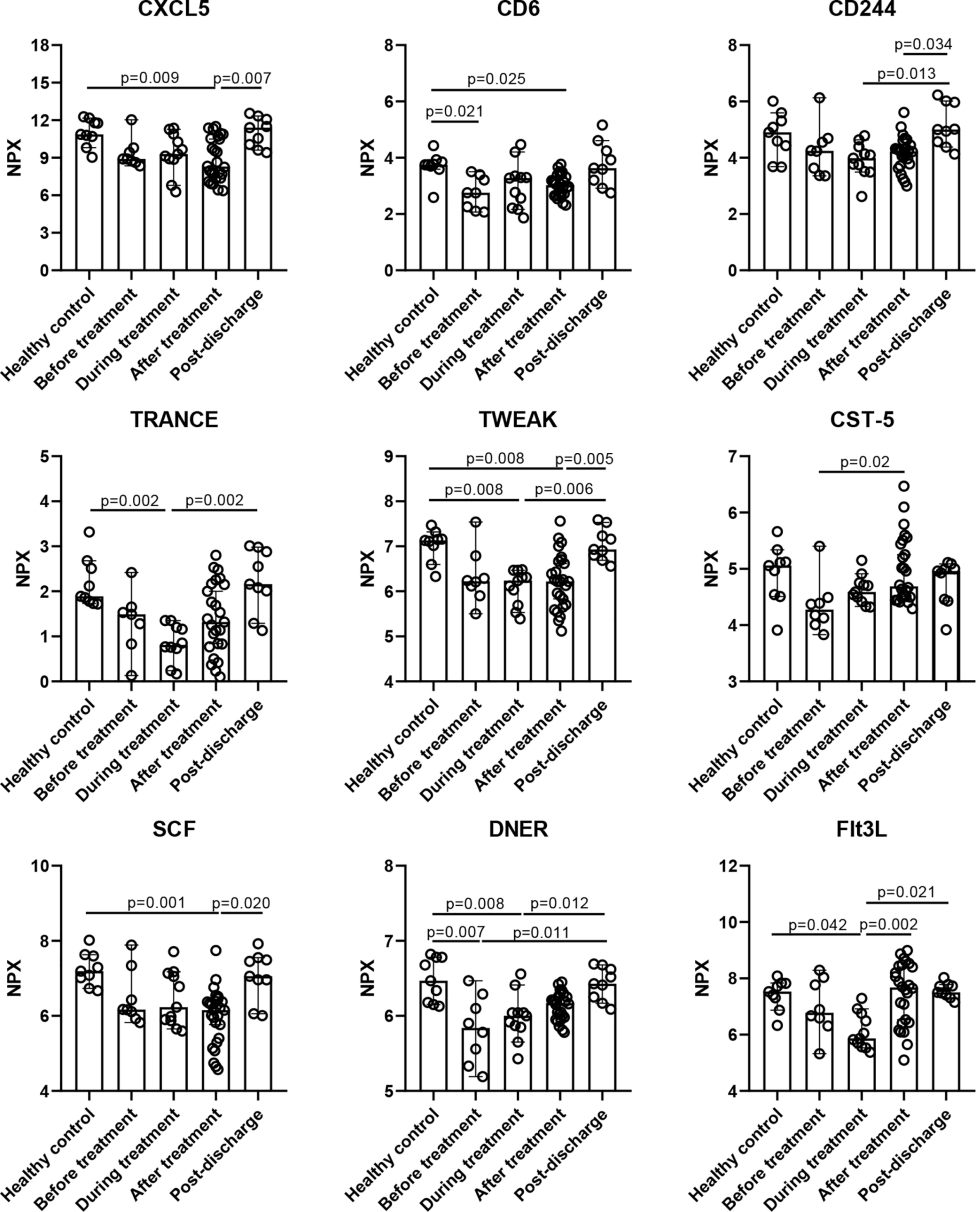
Inflammatory cytokines in cluster 1 were down-regulated during hospitalization. Data are presented as median ± 95% CI. Statistics analysis was performed with Kruskal-Wallis nonparametric method.

To evaluate the effect of MP on inflammatory cytokines in an unbiased manner, we performed an unsupervised clustering analysis that included all the MP patients and healthy control and all the time points using 92 cytokines, and five main clusters emerged, as shown in **Figure 2** and **Table S2**. Cluster 1 comprised CXCL5, MCP-4, FGF-19, IL-12B, CD6, SCF, TWEAK, FGF-21, CD244, TRANCE, CST-5, DNER, TNFB, ST1A1, uPA, CCL28, AXIN1, and Flt3L, and was characterized by reduced cytokine levels at baseline, and among them, the levels of CD6 and DNER were significantly lower than healthy control (**Figure 3**). For most of the cytokines in cluster 1, no significant difference was observed between before and after MP treatment, except that CST-5 increased (p=0.02), the level of which was slightly lower than healthy control at baseline. Besides, four cytokines including CXCL5, CD6, TWEAK, and SCF, were significantly lower than healthy control after MP treatment (p=0.009, p=0.025, p=0.008, and p=0.001, respectively). TRANCE and Flt3L were significantly lower than healthy control during treatment (p=0.002, p=0.042, respectively). These data indicated MP was not effective in rapidly restoring the downregulated cytokines among severe COVID-19 patients. As expected, the level of all the cytokines in cluster 1 reverted to normal over 6 months after discharge.

**Figure 4:**
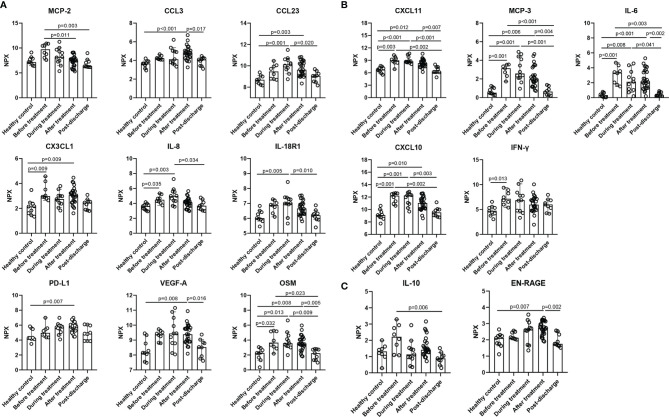
Inflammatory cytokines in cluster 2, cluster 4, and cluster 5 were up-regulated during hospitalization. **(A)** cluster 5, **(B)** cluster 2, **(C)** cluster 4. Data are presented as median ± 95% CI. Statistics analysis was performed with Kruskal-Wallis nonparametric method.

Except for the reduced cluster 1 cytokines, the expression of the other 4 clusters was increased or no difference compared with the healthy control. Cluster 2 was driven by a set of inflammatory markers closely related to CRS. Among them IL-6, CXCL10, CXCL11, and MCP-3 were markedly elevated at baseline, during, and after MP treatment; besides, the differences in all the cytokines between the two time points, before and after MP treatment, were not significant (**Figure 4B**), suggesting MP was not effective in improving these inflammatory markers associated with CRS. Cytokines in cluster 3 such as IL-7 and IL-10RA were similar to healthy control at baseline and did not vary significantly during hospitalization (**Table S2**). Similarly, cytokines in cluster 4 were mildly elevated at baseline and displayed no significant change during hospitalization except EN-RAGE, which is a biomarker of pulmonary injury and associated with the pathogenesis of sepsis-induced ARDS (36), was significantly elevated after MP treatment compared to healthy control (p=0.007, **Figure 4C**). Cluster 5 contained MCP-2, LAP TGF-β1, TNFSF14, CCL4, CCL3, CCL19, TNF, CX3CL1, PD-L1, VEGF-A, OSM, CXCL9, CCL23, IL-8, and IL-18R1 (**Figure 4A**). Among them, IL-8, CX3CL1, and OSM were significantly elevated at baseline, and the latter two remained markedly elevated after MP treatment (p=0.009 and p=0.008, respectively). Besides, the other five cytokines, CCL3, CCL23, VEGF-A, IL-18R1, and the inhibitory immune checkpoint PD-L1, were mildly elevated at baseline and significantly elevated during or after MP treatment. MCP-2 was significantly decreased after MP treatment compared with baseline (p=0.011), but the levels of MCP-2 at the two time points were not significantly different from healthy control.

### Strong Correlations Between CRP and “Core Signature” Cytokines

**Figure 5:**
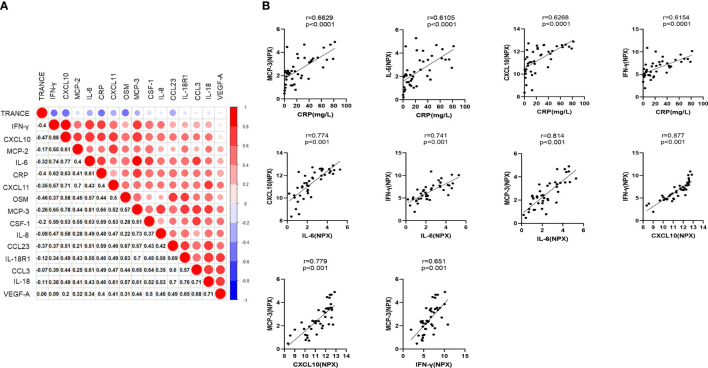
Strong correlations between CRP and “core signature” cytokines. **(A)** Correlation heatmap. The size of circle represents the absolute value of correlation coefficient, correlation heatmap was performed with “corrplot” packages of R studio. **(B)** Cytokines strongly correlated with CRP and each other.

To gain insights into the key immunological features of the severe patients treated with MP, we also correlated the measurements of CRP, d-dimer, Alb, KL-6 and inflammatory cytokines across all sample collection during hospitalization. Except for CRP, there were no significant correlations between D-dimer, Alb, KL-6, and inflammatory cytokines (data not shown). By analyzing the correlation between CRP and inflammatory cytokines, a core signature of this population was primarily defined by four cytokines in cluster 2, which strongly and positively correlated with each other and CRP: MCP-3, IL-6, IFN-γ, and CXCL10 (**Figure 5A, B**). All these cytokines were linked to CRS and COVID-19 prognosis (34, 37). Another cytokine in cluster 2, CXCL11, strongly correlated with CXCL10 (r=0.710, p<0.001) and moderately correlated with CRP (r=0.400, p=0.010), IL-6 (r=0.430, p=0.005), IFN-γ (r=0.570, p<0.001), and MCP-3 (r=0.520, p<0.001), as shown in **Figure 5A**. Besides, other cytokines including IL-8, IL-18R1, VEGF-A, MCP-2, CCL3, CCL23, and OSM in cluster 5, IL-18 and CSF-1 in cluster 4, and TRANCE in cluster 1 moderately correlated with CRP, as shown in **Figure S1**. Notably, MP showed poor efficiency in improving CRP and these “core signature” cytokines (**Figures 1A**, **4B**).

## Discussion

Our longitudinal analyses of severe COVID-19 patients treated with MP revealed the key temporal characteristics of severity biomarkers as well as immune response in this population. We found that a short course of MP was not superior to supportive therapy for severe COVID-19 in improving clinical outcomes and severity biomarkers. Although the severity biomarker, CRP, was markedly decreased after MP treatment, CRP in non-MP group displayed similar kinetics (**Figure 1A**). Besides, no significant difference in the other three severity biomarkers, d-dimer, Alb, and KL-6 between MP and non-MP groups was observed at the same time points during hospitalization (**Figures 1B–D**). Recently it was reported that prolonged MP treatment was effective in reducing CRP compared to supportive therapy, but this benefit seemed to be restricted to within 7 days after admission in their cohort, and at a later stage the levels of CRP were similar between the two groups (18). Reduced mortality has also been reported to be associated with prolonged or high-dose MP regimen (17, 18, 38). Notably, the dosage and duration of MP administration in the literature are highly heterogeneous and might have affected the observed outcome, e.g., a recent clinical trial conducted in Brazil did not find an improvement in mortality with a short course of MP (20). Consistent with this finding, we here showed that a short course of MP could not improve the clinical outcome of severe COVID-19 patients. The regimen of MP in our study was 40 mg or 80 mg daily for 3-5 days. Thus, the results should be interpreted with caution for other regimens of MP. Previous studies have suggested age is an independent risk factor for severe COVID-19 (25). In our cohort, the severe patients enrolled in an unbiased manner indeed represented an older population (**Table 1**).

The markedly elevated inflammatory cytokines at baseline indicate aberrant immune response among severe COVID-19 patients, and a variety of them such as IL-6 are involved in CRS (**Figures 2**, **4B**) (12, 34, 39), which correlates with pulmonary damage, multi-organ injury, and death (40). It was proposed that CRS in COVID-19 was distinct from those associated with sepsis and chimeric antigen receptor T cells, with a prolonged elevation of cytokines over weeks and absence of coordination between them (41). Here, we found a broad spectrum of the cytokines in cluster 2 and 5 were aberrantly elevated at baseline and remained upregulated even after MP treatment (**Figures 4A, B**), likely adding novel evidence to the theory. Besides, we also identified the cytokines in cluster 1, e.g., CD6 was significantly downregulated at baseline (**Figures 2**, **3**). Recent studies showed that CD6 is a co-inhibitory molecule that inhibits T-cell response (42, 43). Thus, a downregulated CD6 at baseline is probably associated with overactivation of T cell response among severe COVID-19 patients.

The “core signature” cytokines, including MCP-3, IL-6, IFN-γ, and CXCL10 in cluster 2, characterized by a strong correlation with CRP and each other, were significantly elevated on admission (**Figure 2** and **4B**). These indicated that “core signature” cytokines as severity biomarkers similar to CRP could be applied to evaluate treatment efficacy. However, we found MP had a limited effect on them as all the “core signature” cytokines remained markedly elevated after MP treatment (**Figure 4B**). Recently it was reported that compared to prednisone and MP, dexamethasone had the highest reduction effect on IL-6, potentially supporting the result of “RECOVERY” trial and our findings (15, 41). IL-8 and OSM in cluster 5 correlate with COVID-19 severity and are considered as predictors for prognosis (39, 44). In MP group, both of them were markedly elevated at baseline and after MP treatment (**Figure 4A**). Besides, we also observed an increased PD-L1 after MP treatment, which might be associated with CRS and CD8+ T-cell exhaustion (45). These data suggested that a short course of MP is not capable to reverse immune dysregulation among severe COVID-19 patients. As mentioned above, some studies also reported lower mortality associated with prolonged or high-dose MP administration (18, 38), it would be interesting to evaluate the effect of these MP regimens on inflammatory cytokines.

It is worth noting that coagulation abnormalities and pulmonary damages among severe COVID-19 patients, presenting as elevated serum d-dimer and KL-6, continued before discharge, when the median of CRP levels was in the normal range (**Figures 1B, D**). These unusual abnormalities may link to post-discharge complications that were reported recently and warrant further research (46). On the other hand, all the aberrant cytokines are restored after discharge more than six months by analysis of 92 cytokines, these data may suggest that immune system had a good prognosis.

There has been a variety of studies regarding the immunological features of COVID-19 (9, 12, 39), but a temporal analysis of patients receiving MP, whose clinical efficacy remains controversial, is still lacking. Thus, our longitudinal study on this population by use of systems biology approach would be a deep expansion. Our study period covers hospitalization and over six months after discharge, rendering a complete picture of the clinical course and cytokine characteristics of COVID-19 patients treated with MP. The limitations of our study include that it is a retrospective, observational study, and the sample size is not large due to a small number of infected COVID-19 cases in Guangzhou although a majority of the local severe patients (88.4%, 38/43) had been included. Indeed, during the study period (by March 13, 2020), the cumulative number of COVID-19 cases in Guangzhou was only 347 according to Guangzhou Municipal Health Commission Report (47). Unfortunately, by now a large, multicenter, randomized trial on clinical efficacy of MP is still lacking.

Taken together, our study evaluated the efficacy of a short course MP among severe COVID-19 patients by use of a variety of well-studied biomarkers and also revealed the “core signature” cytokines, the hallmark of severe COVID-19, as well as the dynamic changes of the immunological features of the population. This would undoubtedly broaden our understanding of the pathogenesis of COVID-19 and provide scientific evidence for future treatment of the highly contagious disease.

## Data Availability Statement

The original contributions presented in the study are included in the article/**Supplementary Material**. Further inquiries can be directed to the corresponding authors.

## Ethics Statement

The studies involving human participants were reviewed and approved by The Ethics Committee of Guangzhou Eighth People’s Hospital, Guangzhou Medical University (No. 202001134). The patients/participants provided their written informed consent to participate in this study.

## Author Contributions

FL, FH, and XLD designed the study. HH collected clinical data. QF and KD performed the experiments. QF performed bioinformatics analyses. QF and KD wrote the manuscript. RH, XZD, YL, YT, WC, and YW collected samples. All authors contributed to the article and approved the submitted version.

## Funding

This work was supported by Guangdong Provincial Department of Science and Technology Fund (No. 2020B1111330002, and 2021A1111110001), Emergency Key Program of Guangzhou Laboratory (EKPG21-29 and EKPG21-31).

## Conflict of Interest

The authors declare that the research was conducted in the absence of any commercial or financial relationships that could be construed as a potential conflict of interest.

## Publisher’s Note

All claims expressed in this article are solely those of the authors and do not necessarily represent those of their affiliated organizations, or those of the publisher, the editors and the reviewers. Any product that may be evaluated in this article, or claim that may be made by its manufacturer, is not guaranteed or endorsed by the publisher.
