# Supplementary material for: Kinetics of Severity Biomarkers and Immunological Features of Methylprednisolone Therapy for Severe COVID-19 Patients
Source: Front Immunol. 2022 Mar 8;13:758946. doi: 10.3389/fimmu.2022.758946 (PMC8957869; doi:10.3389/fimmu.2022.758946)
Supplement: Supplementary file 1 [file DataSheet_1.docx]

Supplementary Material

## 1. Supplementary Figures


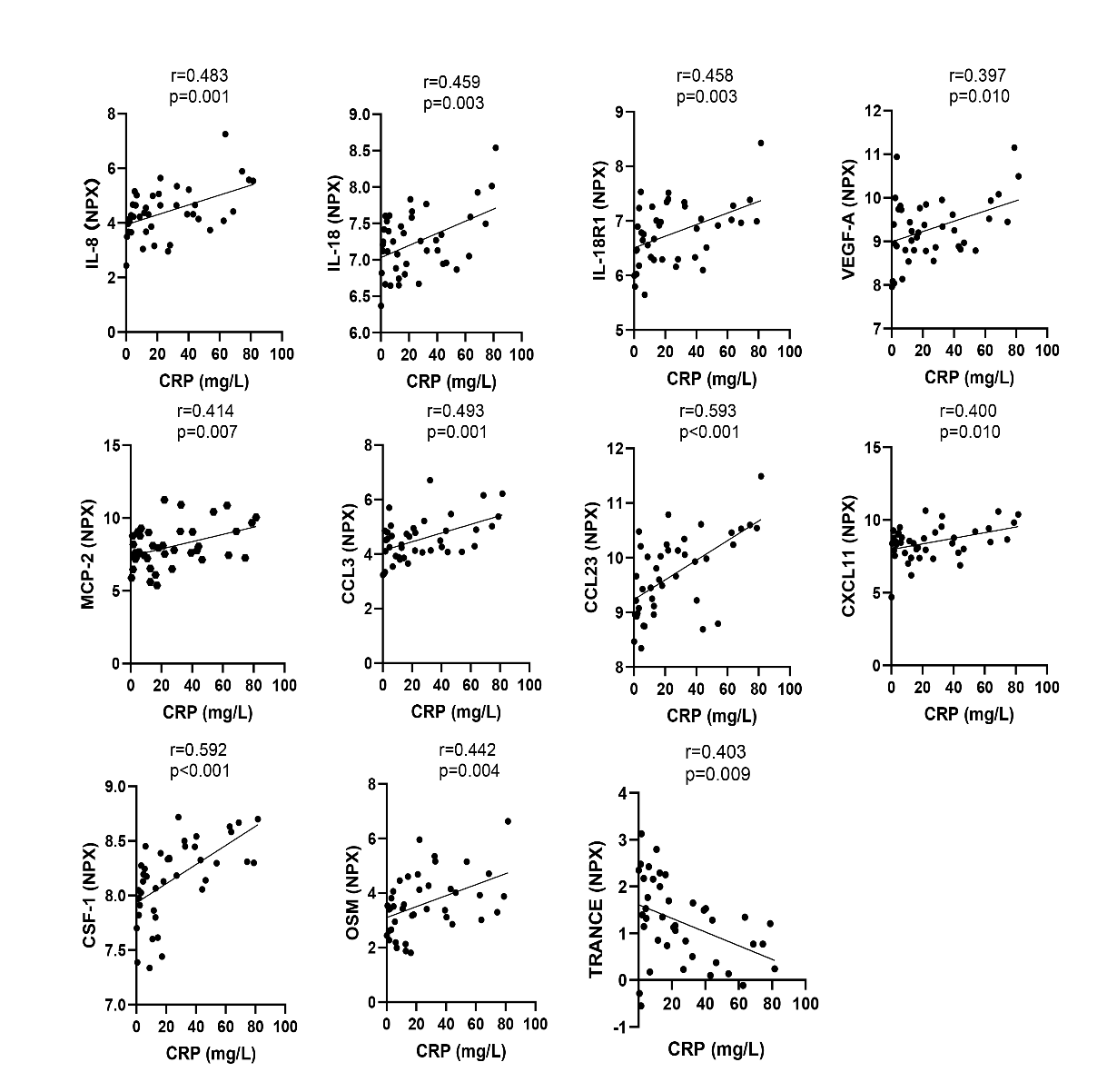


**Supplementary Figure 1.** Moderate correlation between CRP and inflammatory cytokines, including IL-8, IL-18, IL-18R1, VEGF-A, MCP-2, CCL3, CCL23, CXCL11, CSF-1, OSM, and TRANCE.

## 2. Supplementary Tables

**Supplementary Table 1. Inflammatory cytokines for analysis.**

| No. | Short name | Full name | UniProtKB ID | Classification |
| --- | --- | --- | --- | --- |
| 1 | 4EBP1 | Eukaryotic translation initiation  factor 4E-binding protein 1 | Q13541 | translation factor |
| 2 | ADA | Adenosine deaminase | P00813 | deaminase |
| 3 | ARTN | Artemin | Q5T4W7 | neurotrophic factor |
| 4 | AXIN1 | Axin-1 | O15169 | G-protein modulator |
| 5 | BDNF | Brain-derived neurotrophic factor | P23560 | neurotrophic factor |
| 6 | Beta-NGF | Beta-nerve growth factor | P01138 | neurotrophic factor |
| 7 | CASP8 | Caspase-8 | Q14790 | cysteine protease |
| 8 | CCL11 | Eotaxin | P51671 | chemokine |
| 9 | CCL13/MCP4 | C-C motif chemokine 13/Monocyte  chemotactic protein 4 | Q99616 | chemokine |
| 10 | CCL19 | C-C motif chemokine 19 | Q99731 | chemokine |
| 11 | CCL2/MCP1 | C-C motif chemokine 2/Monocyte  chemotactic protein 1 | P13500 | chemokine |
| 12 | CCL20 | C-C motif chemokine 20 | P78556 | chemokine |
| 13 | CCL23 | C-C motif chemokine 23 | P55773 | chemokine |
| 14 | CCL25 | C-C motif chemokine 25 | O15444 | chemokine |
| 15 | CCL28 | C-C motif chemokine 28 | Q9NRJ3 | chemokine |
| 16 | CCL3/MIP1α | C-C motif chemokine 3 | P10147 | chemokine |
| 17 | CCL4 | C-C motif chemokine 4 | P13236 | chemokine |
| 18 | CCL7/MCP3 | C-C motif chemokine 7/Monocyte | P80098 | chemokine |
| 19 | CCL8/MCP-2 | C-C motif chemokine 8/Monocyte  chemotactic protein 2 | P80075 | chemokine |
| 20 | CD244 | Natural killer cell receptor 2B4 | Q9BZW8 | cell adhesion molecule |
| 21 | CD40 | Tumor necrosis factor receptor  superfamily member 5 | P25942 | tumor necrosis factor receptor |
| 22 | CD5 | T-cell surface glycoprotein CD5 | P06127 | oxidase |
| 23 | CD6 | T-cell differentiation antigen CD6 | P30203/  Q8WWJ7 | oxidase |
| 24 | CDCP1 | CUB domain-containing protein 1 | Q9H5V8 | transmembrane glycoprotein |
| 25 | SCF1 | Macrophage colony-stimulating factor 1 | P09603 | cytokine |
| 26 | CST5 | Cystatin-D | P28325 | cysteine protease inhibitor |
| 27 | CX3CL1 | Fractalkine | P78423 | chemokine |
| 28 | CXCL1 | Growth-regulated alpha protein | P09341 | chemokine |
| 29 | CXCL10 | C-X-C motif chemokine 10 | P02778 | chemokine |
| 30 | CXCL11 | C-X-C motif chemokine 11 | O14625 | chemokine |
| 31 | CXCL5 | C-X-C motif chemokine 5 | P42830 | chemokine |
| 32 | CXCL6 | C-X-C motif chemokine 6 | P80162 | chemokine |
| 33 | CXCL9 | C-X-C motif chemokine 9 | Q07325 | chemokine |
| 34 | DNER | Delta and Notch-like epidermal  growth factor-related receptor | Q8NFT8 | growth factor |
| 35 | EN-RAGE/  S100A12 | Protein S100-A12 | P80511 | calmodulin |
| 36 | FGF19 | Fibroblast growth factor 19 | O95750 | growth factor |
| 37 | FGF21 | Fibroblast growth factor 21 | Q9NSA1 | growth factor |
| 38 | FGF23 | Fibroblast growth factor 23 | Q9GZV9 | growth factor |
| 39 | FGF5 | Fibroblast growth factor 5 | P12034/  Q8NF90 | growth factor |
| 40 | GDNF | Glial cell line-derived neurotrophic factor | P39905 | neurotrophic factor |
| 41 | FLT3L | Fms-related tyrosine kinase 3 ligand | P49771 | cytokine |
| 42 | HGF | Hepatocyte growth factor | P14210 | growth factor |
| 43 | IFN-γ | Interferon gamma | P01579 | interferon superfamily |
| 44 | IL-10 | Interleukin-10 | P22301 | interleukin superfamily |
| 45 | IL-10RA | Interleukin-10 receptor subunit alpha | Q13651 | defense/immunity protein |
| 46 | IL-10RB | Interleukin-10 receptor subunit beta | Q08334 | defense/immunity protein |
| 47 | IL-12B | Interleukin-12 subunit beta | P29460 | interleukin superfamily |
| 48 | IL-13 | nterleukin-13 | P35225 | interleukin superfamily |
| 49 | IL-15RA | Interleukin-15 receptor subunit alpha | Q13261 | cytokine receptor |
| 50 | IL-17A | nterleukin-17A | Q16552 | interleukin superfamily |
| 51 | IL-17C | Interleukin-17C | Q9P0M4 | chemokine |
| 52 | IL-18 | Interleukin-18 | Q14116 | interleukin superfamily |
| 53 | IL-18R1 | Interleukin-18 receptor 1 | Q13478 | type I cytokine receptor |
| 54 | IL-1α | Interleukin-1 alpha | P01583 | interleukin superfamily |
| 55 | IL-2 | Interleukin-2 | P60568 | interleukin superfamily |
| 56 | IL-20 | Interleukin-20 | Q9NYY1 | interleukin superfamily |
| 57 | IL-20RA | Interleukin-20 receptor subunit alpha | Q9UHF4 | defense/immunity protein |
| 58 | IL-22RA1 | Interleukin-22 receptor subunit alpha-1 | Q8N6P7 | defense/immunity protein |
| 59 | IL-24 | Interleukin-24 | Q13007 | interleukin superfamily |
| 60 | IL-2RB | Interleukin-2 receptor subunit beta | P14784 | type I cytokine receptor |
| 61 | IL-33 | Interleukin-33 | O95760 | interleukin superfamily |
| 62 | IL-4 | Interleukin-4 | P05112 | interleukin superfamily |
| 63 | IL-5 | Interleukin-5 | P05113 | interleukin superfamily |
| 64 | IL-6 | Interleukin-6 | P05231 | interleukin superfamily |
| 65 | IL-7 | Interleukin-7 | P13232 | interleukin superfamily |
| 66 | IL-8/CXCL8 | Interleukin-8 | P10145 | chemokine |
| 67 | KITLG/SCF | Kit ligand/Stem cell factor | P21583 | cell adhesion molecule |
| 68 | LIF | Leukemia inhibitory factor | P15018 | cytokine |
| 69 | LIF-R | Leukemia inhibitory factor receptor | P42702 | cytokine |
| 70 | LTA/TNFB | Lymphotoxin-alpha/TNF-beta | P01374 | tumor necrosis factor  family member |
| 71 | MMP1 | Interstitial collagenase | P03956 | extracellular matrix  organization |
| 72 | MMP10 | Stromelysin-2 | P09238 | extracellular matrix  organization |
| 73 | NRTN | Neurturin | Q99748 | neurotrophic factor |
| 74 | NTF3/NT3 | Neurotrophin-3 | P20783 | neurotrophic factor |
| 75 | OSM | Oncostatin-M | P13725 | interleukin superfamily |
| 76 | PDL1 | Programmed cell death 1 ligand 1 | Q9NZQ7 | immunoglobulin receptor  superfamily |
| 77 | PLAU/uPA | Urokinase-type plasminogen activator | P00749 | serine protease |
| 78 | SIRT2 | NAD-dependent protein deacetylase sirtuin-2 | Q8IXJ6 | chromatin/chromatin-binding  protein |
| 79 | SLAMF1 | Signaling lymphocytic activation molecule | Q13291 | cell adhesion molecule |
| 80 | STAMBP | STAM-binding protein | O95630 | cytokine |
| 81 | SULT1A1/ST1A1 | Sulfotransferase 1A1 | P50225 | transferase |
| 82 | TGF-α | Transforming growth factor alpha | P01135 | growth factor |
| 83 | TGFβ1/  LAP-TGFβ1 | Latency-associated peptide Transforming  growth factor beta-1 | P01137 | growth factor |
| 84 | TNF | Tumor necrosis factor | P01375 | tumor necrosis factor  family member |
| 85 | TNFRSF11B/OPG | Tumor necrosis factor receptor superfamily  member 11B/Osteoprotegerin | O00300 | tumor necrosis factor  receptor |
| 86 | TNFRSF9 | Tumor necrosis factor receptor  superfamily member 9 | Q07011 | tumor necrosis factor  receptor |
| 87 | TNFSF10/TRAIL | Tumor necrosis factor ligand superfamily  member 10/TNF-related apoptosis-inducing  ligand | P50591 | tumor necrosis factor  family member |
| 88 | TNFSF11/TRANCE | Tumor necrosis factor ligand  superfamily member 11 | O14788 | tumor necrosis factor  family member |
| 89 | TNFSF12/TWEAK | Tumor necrosis factor ligand  superfamily member 12 | O43508 | tumor necrosis factor  family member |
| 90 | TNFSF14 | Tumor necrosis factor ligand  superfamily member 14 | O43557 | tumor necrosis factor  family member |
| 91 | TSLP | Thymic stromal lymphopoietin | Q969D9 | cytokine |
| 92 | VEGF-A | Vascular endothelial growth factor A | P15692 | growth factor |

**Supplementary Table 2.** **Clusters of inflammatory cytokines in MP group.**

| Clusters | Inflammatory cytokines | Longitudinal trends |
| --- | --- | --- |
| Cluster 1 | CXCL5, MCP-4, FGF-19, IL-12B, CD6, SCF, TWEAK, FGF-21, CD244, TRANCE, CST-5, DNER, TNFB, ST1A1, uPA, CCL28, AXIN1, Flt3L | Downregulated at baseline and after MP treatment |
| Cluster 2 | CXCL11, MCP-3, IL-6, CXCL10, IFN-γ | Significantly upregulated at baseline and after MP treatment |
| Cluster 3 | IL-7, IL-10RA, IL-17C, NRTN, IL-20RA, IL-15RA, SLAMF1, OPG, LIF-R, CD8A, CCL11, IL-13, LIF, IL-17A, CCL25, ARTN, FGF-23, IL-22RA, CXCL1, MMP-10, NT-3, β-NGF, ADA, CXCL6, CASP-8, IL-20, CD40, STAMBP, IL-1α, IL-10RB, CD5, TRAIL, TNFRSF9 | Similar to the level of healthy control during hospitalization |
| Cluster 4 | IL-24, IL-10, CCL20, IL-2, TSLP, EN-RAGE, 4E-BP1, SIRT2, IL-18, HGF, CSF-1, TGF-α， MMP-1, IL-4, GDNF, IL-2RB, CDCP1, MCP-1, FGF-5, IL-33, IL-5 | Mildly upregulated at baseline and did not vary significantly during hospitalization |
| Cluster 5 | MCP-2, LAP TGF-β1, TNFSF14, CCL4, CCL3, CCL19, TNF, CX3CL1, PD-L1, VEGF-A, OSM, CXCL9, CCL23, IL-8, IL-18R1 | Mildly or significantly upregulated at baseline and remained upregulated after MP treatment |
